# Psychological Flexibility as a Resilience Factor in Individuals With Chronic Pain

**DOI:** 10.3389/fpsyg.2019.02016

**Published:** 2019-09-03

**Authors:** Charlotte Gentili, Jenny Rickardsson, Vendela Zetterqvist, Laura E. Simons, Mats Lekander, Rikard K. Wicksell

**Affiliations:** ^1^Functional Area Medical Psychology, Functional Unit Behavior Medicine, Karolinska University Hospital, Stockholm, Sweden; ^2^Department of Clinical Neuroscience, Karolinska Institutet, Stockholm, Sweden; ^3^Department of Neuroscience, Uppsala University, Uppsala, Sweden; ^4^Department of Anesthesiology, Perioperative and Pain Medicine, Stanford University School of Medicine, Palo Alto, CA, United States

**Keywords:** chronic pain, psychological flexibility, resilience, avoidance, values

## Abstract

Resilience factors have been suggested as key mechanisms in the relation between symptoms and disability among individuals with chronic pain. However, there is a need to better operationalize resilience and to empirically evaluate its role and function. The present study examined psychological flexibility as a resilience factor in relation to symptoms and functioning among 252 adults with chronic pain applying for participation in a digital ACT-based self-help treatment. Participants completed measures of symptoms (pain intensity, and anxiety), functioning (pain interference and depression), as well as the hypothesized resilience factor psychological flexibility (measured as avoidance, value obstruction, and value progress). As expected, symptoms, functioning and resilience factors were significantly associated. Hierarchical linear regression analyses showed that psychological flexibility significantly contributed to the prediction of pain interference and depression when adjusting for age, pain and anxiety. Also, participants with low levels of psychological flexibility were more likely to be on sick leave. Furthermore, a series of multiple mediation analyses showed that psychological flexibility had a significant indirect effect on the relationship between symptoms and functioning. Avoidance was consistently shown to contribute to the indirect effect. Results support previous findings and suggest the importance of psychological flexibility as a resilience factor among individuals with chronic pain and anxiety.

## Introduction

Resilience – the ability to adapt and function well despite significant strain – has gained increasing attention in the field of chronic pain management. Turk et al. (2008) concluded that “Living with chronic pain requires considerable emotional resilience and tends to deplete emotional reserve.” Patients often report “being stuck” or “putting life on hold” as a consequence of chronic pain, which corresponds with data showing that pain interference rather than pain intensity, predicts levels of functioning (Kemani et al., 2016). Why some patients continue to function well in day to day life after the onset of chronic pain, and others do not, is yet unclear (Reid et al., 2011; Hauser et al., 2014; Vervoort et al., 2014; Goubert and Trompetter, 2017).

Resilience is commonly defined as overcoming adversity or “effective functioning, despite the exposure to stressful circumstances, and/or internal distress” (Sturgeon and Zautra, 2013). However, current definitions of resilience provide limited information on *how* a person recovers or maintain functioning during and following difficult life events. The concept of resilience sometimes reflects a deterministic view where resilience factors are relying on personality traits, biology, social support, past experiences, and/or innate properties such as sex. Resilience may alternatively, and in accordance with Goubert and Trompetter (2017), be conceptualized as a contextual behavioral factor, and defined as the ability to continuously engage in meaningful activities that promote current and future quality of life and health, in the presence of pain and distress (Goubert and Trompetter, 2017). This conceptualization of resilience is closely related to the construct *psychological flexibility*, defined as the ability to act in alignment with values and long-term goals in the presence of inner discomfort such as pain and distress and has been suggested as a key factor in maintaining or improving functioning among individuals with chronic pain (Hayes et al., 1999, 2006).

Lack of psychological flexibility, or psychological *in*flexibility, is commonly displayed as *avoidance* of stimuli and situations associated with pain, and related distress. From a learning perspective, avoidance is an operant under contextual control, meaning that a behavior is influenced by environmental factors (internal and external) preceding the behavior, and/or acting as consequences of that same behavior. Avoidance behaviors may be reinforced – for example resulting in short term reductions of pain or discomfort – and therefore sustained and used in similar contexts. Over time, such operant behaviors may become a default strategy in situations perceived as threatening, with increasing difficulties for the individual to respond differently (Vowles et al., 2007). Due to contextual factors, the behavior repertoire becomes increasingly narrow and less flexible. This behavior pattern is normally not associated with a corresponding decrease in pain or distress, but rather a life less stimulating and active. Avoidance may take many different forms, such as not engaging in social or physical activities, excessive opioid use (overt behaviors), thinking about situations associated with pain or refraining from planning future events (covert behaviors).

*Value orientation* is another key aspect of psychological flexibility, that can be defined as verbal guidelines that function to initiate and maintain behavior over time, also without the presence of obvious reinforcers (Hayes et al., 1999; Hayes, 2012). For example, clarifying the value (“being an attentive parent”) associated with an operant (“playing in the park”) can alter the context and thereby increase the likelihood that this behavior is initiated and/or maintained also in the context of potentially interfering pain. For chronic pain patients, value-based behaviors have been associated with higher levels of functioning and improved mood (Vowles et al., 2014) and adding a value aspect in an experimental intervention has been shown to elevate pain tolerance (Branstetter-Rost et al., 2009). Value orientation usually comprise components such as clarifying and engaging in value-based behaviors, including effectively dealing with obstacles to value-based behaviors (Smout et al., 2014).

The aim of the present study was to examine the role and function of psychological flexibility, assessed as avoidance, value obstruction, and value progress as resilience factors in a sample of individuals with chronic pain. More specifically, analyses were conducted to (1) broadly characterize the relationship between symptoms, functioning and psychological flexibility, (2) assess the amount of variance in functioning (pain interference and depression) explained by psychological flexibility, (3) explore low vs. high psychological flexibility as a potential risk/resilience factor for self-reported sick leave and opioid use, and (4) examine the indirect effects of psychological flexibility in the relationship between symptoms and functioning.

## Materials and Methods

### Data Collection

This study used a cross-sectional design, with data from baseline assessments for participants applying for internet-delivered Acceptance and Commitment Therapy for chronic pain (ClinicalTrials.gov identifier: NCT03105908 and NCT03344926). Participants were recruited via ads in newspapers and social media between January 30, 2017 and January 31, 2018. All questionnaires and demographic questions were completed online in a secure web platform. Participants provided written informed consent prior to enrollment in the study, the study was approved by the Regional Ethics Committee and followed the Helsinki declaration.

Eligibility criteria were pain duration over 6 months, age 18 years or older, ability to read and write in Swedish and completion of all assessments.

### Measurements

Demographic variables included age, sex and educational level.

#### Symptom Variables

*Pain variables* included *pain intensity* – current and average in the past week – measured with a numeric rating scale (NRS, 0 = no pain at all, 10 = worst pain imaginable), *pain duration* (self-reported in years), and *pain location* (self-reported descriptions of localization).

To account for some of the complexity of symptoms commonly co-occuring with chronic pain, the present study used *anxiety* as measured with Generalized Anxiety Disorder-7 (GAD-7) (Spitzer et al., 2006) as a proxy for distress symptoms such as strain, worry, and restlessness.

GAD-7 measures the frequency of anxiety symptoms during the last 2 weeks are scored on a four-point Likert scale (0 = Not at all, 3 = Every day). A total score of 10 was chosen as cut-off for anxiety problems, in accordance with guidelines (Spitzer et al., 2006). Internal consistency (Cronbach’s Alpha) was 0.92 in the validation study (Spitzer et al., 2006), and 0.88 in the present dataset. GAD-7 is validated for chronic pain patients with migraine (Seo and Park, 2015a).

#### Functioning

*Pain interference* was measured with the pain interference index (PII), a brief self-report questionnaire assessing the influence of pain on behavior, or to what extent pain interferes with daily functioning (Kemani et al., 2016). Six items are rated on a seven-point Likert scale (0 = Not at all, 6 = Completely). Internal consistency (Cronbach’s Alpha) in the present sample was 0.85, which corresponds to the alpha from the original validation study, which was performed on a chronic pain sample (Kemani et al., 2016).

Level of *depression* was measured using the Patient Health Questionnaire 9 items (PHQ-9) (Kroenke et al., 2001). The frequency of depressive symptoms occurring during the past 2 weeks are rated on a four-point Likert scale from 0 = Not at all to 3 = Nearly every day. Internal consistency (Cronbach’s Alpha) in the present sample was 0.81, and in the original validation studies Alpha varied from 0.86 to 0.89 (Kroenke et al., 2001). PHQ-9 has been validated for chronically ill patients (Wu, 2014) and migraine patients (Seo and Park, 2015b). PHQ-9 was used as a measure of functioning in this study, with the theoretical assumption that depressive disorder is a result of a series of behaviors, not merely the occurrence of symptoms.

*Sick leave* during the past 2 months was assessed using self-report and classified as temporary, permanent, or none. Due to a large overlap between temporary and permanent sick leave, the variable was transformed to a binary variable (sick leave/no sick leave).

Data on *opioid use* was collected via self-report. Participants listed all current medications, which then were classified according to the Anatomical Therapeutic Chemical classification system by an anesthesiologist. Participants with opioids in their list were coded as “currently taking opioids” and participants without opioids in their list were coded as “currently not taking opioids.”

#### Psychological Flexibility

In the present study, two aspects of psychological flexibility were assessed: avoidance and values orientation.

*Avoidance* was measured using a subscale of Psychological Inflexibility in Pain Scale (PIPS) (Wicksell et al., 2008b). The avoidance subscale consists of 8 items, rated on a seven-point Likert scale from 1 = never true, to 7 = always true. The avoidance subscale has consistently been shown to be a robust and valid measure in chronic pain samples (Wicksell et al., 2010a; Barke et al., 2015). In the present sample internal consistency was 0.91, which corresponds with an alpha of 0.89 in the original validation study (Wicksell et al., 2008b).

*Values orientation* was assessed using the subscales *value progress* and *value obstruction* from the Valuing Questionnaire (VQ) (Smout et al., 2014). Items are rated on a seven-point Likert-scale ranging from 0 = not at all true, to 6 = completely true. Higher scores on the progress subscale indicate greater progress toward value-based behavior, while higher scores on the obstruction subscale indicate greater obstruction to value-based behavior. The two-factor solution has shown adequate model fit and strong internal consistency (Cronbach’s alpha = 0.87) (Smout et al., 2014). In the present study Cronbach’s Alpha was 0.83 for value progress and 0.76 for value obstruction. The Valuing Questionnaire has been validated for use with chronic pain samples (Carvalho et al., 2018).

Avoidance and value obstruction are both negatively valenced measures (measuring the occurrence of something negative) while value progress is positively valenced (measuring the presence of something positive). However, both are important aspects of the psychological flexibility model (McCracken and Morley, 2014).

### Participant Characteristics

The sample consists of adults with chronic pain applying for participation in a digital ACT-based self-help treatment. The sign-up-process for the clinical trial was initiated by 266 persons, of which 253 completed the assessment. One individual was excluded due to pain duration <6 months. Thus, data from 252 participants was used in the analyses.

Participants were predominantly female (81%, *n* = 204) and born in Sweden (90%, *n* = 226) with a mean age of 47.4 years (SD 11.5, range 18–70). Two thirds (66%, *n* = 166) of the sample had some level of university education (>12 years of education), nearly one third (30%, *n* = 75) had completed upper secondary school (12 years), and a few participants (4%, *n* = 11) had completed only compulsory school (9 years). Occupational status varied, with 31% (*n* = 79) working full-time, 25% (*n* = 62) part-time, 25% (*n* = 63) being on temporary sick leave, and 23% (*n* = 59) on permanent disability.

Pain duration was on average 18.2 years (SD 12.5, range 0.5–57), and the participants reported that last week’s mean pain intensity was 6.6 (SD 1.7, range 1–10). Most individuals had multiple pain locations (88%, *n* = 222), and the most common pain locations were: back (75%, *n* = 188), neck (64%, *n* = 160), and lower extremities (64%, *n* = 160). Half of the sample experienced headaches (50%, *n* = 125) and 40% (*n* = 101) experienced generalized pain. Primary pain diagnoses were classified by an anesthesiologist as nociceptive (e.g., spinal disc hernia, and rheumatoid arthritis) for 37% (*n* = 93), as nociplastic (e.g., fibromyalgia and CRPS) for 17% (*n* = 44), neuropathic (e.g., trigeminal neuralgia and nerve damage) for 8% (*n* = 20), and headaches (e.g., migraine and Horton’s) for 8% (*n* = 19). A fifth (19%, *n* = 47) had no diagnosis and 12% had mixed or unclassifiable pain diagnosis. Furthermore, more than half (54%, *n* = 157) scored above cut-off for depression, and one in four (25%, *n* = 64) above cut-off for anxiety.

### Statistical Analyses

Analyses were computed using SPSS version 25 and STATA version 15. The dataset was complete, and no imputation strategies were needed. In all analyses, statistical significance was set to a conservative level of *p* < 0.01, except in the criteria for inclusion of covariates where *p* < 0.05 was used.

To determine the adequate sample size for mediation and regression analyses, a power analysis was conducted using the G^∗^power software (Faul et al., 2009). Assuming a medium effect size (*f*^2^) of 0.15, an alpha of 0.01, a power level of 0.80 and a total of 6 predictors, the power analysis suggested a minimum of 109 participants.

Initial analyses were conducted to broadly characterize the relationships between the variables using descriptive statistics and Pearson’s product moment correlations (*r*) (Field, 2013).

A series of hierarchical linear regression analyses were performed to investigate the amount of variance explained by psychological flexibility (avoidance, value obstruction, and value progress) in pain interference and depression. Demographic variables were entered as step 1, symptoms were added as step 2, and psychological flexibility as step 3. For each analysis, only variables having a significant bivariate correlation (*p* < 0.05) with the dependent variable were included.

The relationships between levels of psychological flexibility and the risk of sick leave and opioid use were analyzed using maximum likelihood logistic regression models. We estimated risks, or odds ratios (OR), for sick leave and opioid use (dependent variables) with each respective measure of psychological flexibility (avoidance, value obstruction, and value progress) as independent variables. The independent variables were categorized as low (first quartile), medium (second and third) and high (fourth) of the continuous distributions in line with recommendations from van Kuijk et al. (2019), as the association between predictors and outcomes was not linear and the assumption of non-additivity between different predictors and covariates was not met (van Kuijk et al., 2019). Age and pain intensity showed bivariate correlations with the dependent variables and were therefore used as covariates in these analyses.

A series of analyses of indirect effects using PROCESS for SPSS were conducted to evaluate the importance of psychological flexibility (avoidance, value obstruction, and value progress) for the relationships between predictors (pain and anxiety) and dependent variables (pain interference and depression). In all analyses the influence of age was adjusted for (covariate). Four models were analyzed, with each predictor and dependent variable, and with multiple mediators (PROCESS model #4). PROCESS is a bootstrapping method in which samples of the original size, drawn from the original data, are generated (Hayes and Rockwood, 2017). The total effect (*c*) is comprised of the direct effect (*c*′) and the indirect effect (*ab*). Thus, the indirect effect represents the part of the relation between the predictor and the dependent variable that can be explained by the proposed mediator. The mean value for the *ab* product across the bootstrapped samples provided a point estimate of the indirect effect. Confidence intervals (CI) were derived from the obtained distribution of *ab*, using a 99% CI level representing a significance level of *p* > 0.01. If lower and upper bounds did not contain zero, the indirect effect was significant at the specified level. Each analysis was based on 5000 bootstrapped samples, as suggested by Preacher and Hayes (2008).

## Results

Mean, standard deviations, and range for all self-report measures are reported in Table 1.

**TABLE 1:** Self-report measures: Means, standard deviations, and range.

**Measures**	**Mean (SD)**	**Range (possible range)**
Pain intensity average	6.6 (1.7)	1–10 (0–10)
Anxiety	7.1 (5.1)	0–21 (0–21)
Pain interference	23.5 (8.3)	1–36 (0–36)
Depression	11.3 (5.7)	0–26 (0–27)
Avoidance	35.9 (9.7)	8–56 (8–56)
Value obstruction	14.2 (6.8)	0–30 (0–30)
Value progress	14.1 (6.7)	0–30 (0–30)

### Bivariate Correlations Between Symptoms, Functioning, and Psychological Flexibility

Strong positive correlations were found between avoidance and pain interference (*r* = 0.668), avoidance and depression (*r* = 0.514), value obstruction and depression (*r* = 0.522) as well as between anxiety and depression (*r* = 0.663). Pairwise correlations between all variables are shown in Table 2.

**TABLE 2:** Correlations between all variables.

**Variable**	**Sex^1^**	**Edu^1^**	**Dur**	**Intensity**	**Anx**	**Interfer**	**Depr**	**Sick^1^**	**Opioid^1^**	**Avoid**	**Obstr**	**Progress**
Age	–0.099	–0.060	0.300^∗∗^	0.042	–0.304^∗∗^	–0.166^∗∗^	–0.245^∗∗^	0.159^∗^	0.012	−0.141^∗^	–0.266^∗∗^	0.127^∗^
Sex^1^	–	0.042	–0.006	0.081	0.101	0.067	–0.004	0.036	–0.108	–0.041	–0.024	0.004
Education^1^		–	0.022	–0.124	–0.023	–0.028	–0.034	–0.053	–0.029	–0.084	–0.057	0.033
Symptoms (predictors)												
Pain duration			–	0.110	–0.045	0.010	–0.022	0.051	0.077	–0.045	–0.093	0.037
Pain intensity				–	0.180^∗∗^	0.356^∗∗^	0.191^∗∗^	–0.002	0.141^∗^	0.266^∗∗^	0.101	–0.018
Anxiety					–	0.387^∗∗^	0.663^∗∗^	–0.081	–0.039	0.342^∗∗^	0.555^∗∗^	–0.309^∗∗^
Functioning (dependent variables)												
Pain interference						–	0.594^∗∗^	0.216^∗∗^	0.180^∗∗^	0.668^∗∗^	0.439^∗∗^	–0.311^∗∗^
Depression							–	0.091	0.084	0.514^∗∗^	0.522^∗∗^	–0.422^∗∗^
Sick leave^1^								–	0.178^∗∗^	0.184^∗∗^	0.013	–0.188^∗∗^
Opioid use^1^									–	0.121	–0.006	–0.074
Psychological flexibility (Independent variables)												
Avoidance										–	0.465^∗∗^	–0.425^∗∗^
Value obstruction											–	–0.386^∗∗^
Value progress												–

*^∗^*p* < 0.05, ^∗∗^*p* < 0.01. ^1^Spearman correlation used for categorical variables. Edu, education; Dur, pain duration; Intensity, pain intensity; Anx, anxiety; Interfer, pain interference; Depr, depression; Avoid, avoidance; Obstr, obstruction.*

### Amount of Variance in Functioning Explained by Psychological Flexibility

Hierarchical regression analyses were conducted to evaluate the amount of variance explained by psychological flexibility in the two dependent variables *pain interference* and *depression*. Age had a significant positive bivariate correlation with sick-leave and a significant negative bivariate correlation with pain interference and depression and was therefore entered as step 1.

#### Pain Interference

Psychological flexibility explained a significant amount of variance in pain interference (*r*^2^ change = 0.44, *p* < 0.0001), when adjusting for the influence of pain and anxiety (*r*^2^ = 0.27, *p* < 0.0001). Of the psychological flexibility variables solely avoidance showed a significant – and positive – beta value (*b* 0.52, *p* < 0.0001).

#### Depression

In depression, psychological flexibility explained a significant amount of variance (*r*^2^ change = 0.11, *p* < 0.0001) when adjusting for the influence of pain and anxiety. Avoidance had a significant positive beta coefficient and value progress had a significant negative beta coefficient in the model. Results from the hierarchical regression analyses are presented in Table 3.

**TABLE 3:** Hierarchical linear regressions: the influence of psychological flexibility on functioning.

**Dependent variable**	**Step**	**Predictors**	***R*^2^**	***R*^2^ Change**	***F* Change (*df*)**	**Sig. *F* Change**	**Standardized Beta with all variables entered**		
							
							**β**	***t***	**Sig.**
Pain interference	1	Demographics	0.03	0.03^∗∗^	7.10 (1, 250)	0.008			
		Age					–0.04	–0.85	0.395
	2	Symptoms	0.24	0.21^∗∗^	34.9 (2, 248)	<0.0001			
		Pain intensity					0.19^∗∗^	4.01	<0.0001
		Anxiety					0.10	1.78	0.077
	3	Psy flex	0.51	0.27^∗∗^	45.55 (3, 245)	<0.0001			
		Avoidance					0.52^∗∗^	9.58	<0.0001
		Obstruction					0.11	1.83	0.068
		Progress					–0.01	–0.13	0.895
Depression	1	Demographics	0.06	0.06^∗∗^	16.03 (1, 250)	<0.0001			
		Age						–0.62	0.533
	2	Symptoms	0.45	0.39^∗∗^	86.91 (2, 248)	<0.0001			
		Pain intensity					0.03	0.69	0.493
		Anxiety					0.48^∗∗^	8.96	<0.0001
	3	Psy flex	0.55	0.11^∗∗^	19.54 (3, 245)	<0.0001			
		Avoidance					0.24^∗∗^	4.63	<0.0001
		Obstruction					0.08	1.44	0.152
		Progress					–0.14^∗∗^	–2.75	0.006

*Psy flex, psychological flexibility. ^∗∗^*p* < 0.01.*

### Odds for Sick Leave and Opioid Use in Individuals With Low vs. High Psychological Flexibility

Logistic regression analyses were conducted to evaluate the odds for sick leave and opioid use in individuals with low and high psychological flexibility.

#### Sick Leave

The odds of being on sick leave was four times lower in the low value progress group compared to the high value progress group (OR 0.25, *p* = 0.001). For avoidance, the high avoidance group had lower odds for being on sick leave (OR 5.23, *p* < 0.0001) compared to the low avoidance group. For different levels of value obstruction there were no significant differences in odds for sick leave.

#### Opioid Use

For opioid use, no significant difference in odds between high and low avoidance, value obstruction, or value progress were found. Results from the logisitic regression analyses are presented in Table 4.

**TABLE 4:** Odds ratios for sick leave and opioid use, respectively, for low, medium and high levels of psychological flexibility, with age and pain intensity as covariates.

**Dependent**									
**variable**	**Predictor**	**Low (ref. odds)**		**Medium**			**High**		
				
		**Yes/No**	**OR**	**Yes/No**	**OR**	**OR 99% CI**	**Yes/No**	**OR**	**OR, 99% CI**
Sick leave									
	Avoidance	15/45	1.0	64/64	3.54^∗∗^	1.40–8.95	35/29	5.23^∗∗^	1.74–15.74
	Obstruction	28/35	1.0	56/77	1.24	0.51–2.97	30/26	2.56	0.81–8.25
	Progress	43/27	1.0	51/74	0.37^∗∗^	0.16–0.85	20/37	0.25^∗∗^	0.09–0.72
Opioid use									
	Avoidance	16/44	1.0	40/88	1.30	0.52–3.26	28/36	2.30	0.79–6.70
	Obstruction	23/40	1.0	43/90	0.97	0.39–2.38	18/38	0.97	0.29–3.21
	Progress	26/44	1.0	41/84	0.78	0.34–1.79	17/40	0.60	0.21–1.67

*^∗∗^*p* < 0.01.*

### The Indirect Effect of Psychological Flexibility in the Relationship Between Symptoms and Functioning

The bootstrap method (PROCESS) with *n* = 5000 bootstrap resamples and 99% bias-corrected and accelerated confidence intervals was used in a series of analyses conducted to evaluate the indirect effects of psychological flexibility (M1 = avoidance, M2 = progress, and M3 = obstruction) on the relationship between symptoms (average pain intensity and anxiety) and functioning (pain interference and depression).

**FIGURE 1:**
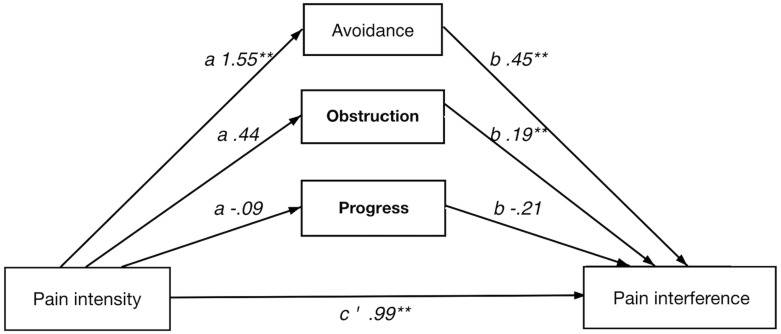
Direct and indirect effect of pain intensity on pain interference.

**FIGURE 2:**
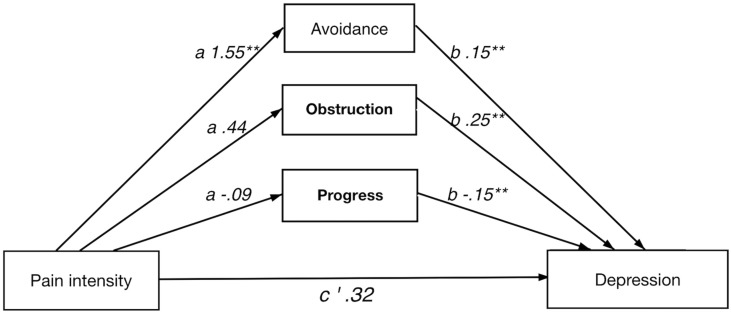
Direct and indirect effect of pain intensity on depression.

**FIGURE 3:**
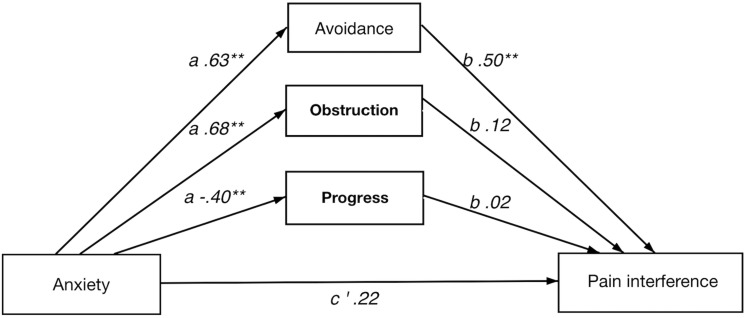
Direct and indirect effect of anxiety on pain interference.

**FIGURE 4:**
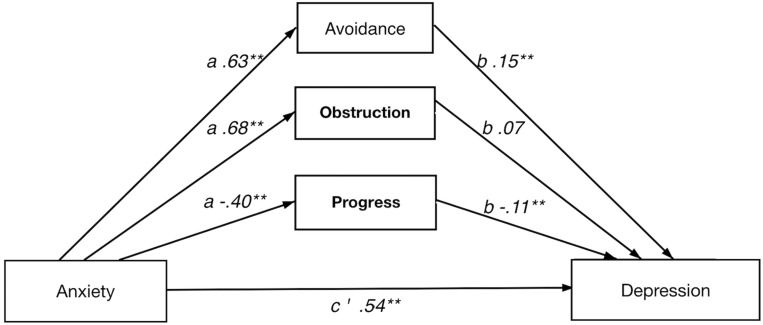
Direct and indirect effect of anxiety on depression.

In short, all four multiple indirect effect models, with pain intensity/anxiety (*x*) as predictors and pain interference/depression (*y*) as dependent variables, showed a significant total indirect effect of psychological flexibility, i.e., the combined indirect effects of avoidance (M1), progress (M2), and obstruction (M3). Results from the analyses of indirect effects are summarized in Table 5. Detailed results for each model are presented below and in Figures 1–4.

**TABLE 5:** Total, direct and indirect effect of symptoms on pain interference and depression using psychological flexibility as indirect effect.

							**Indirect effect**		
							
			***a* path**	***b* path**	**Total**	**Direct**			
***X***	***Y***	***m***	**coefficient**	**coefficient**	**effect (c)**	**effect (c′)**		**CI (99%)**	
								
							**Effect (SE)**	**LLCI**	**ULCI**
Pain intensity	Pain interference	Psy flex			1.77^∗∗^	0.99^∗∗^	0.78^∗∗^(0.20)	0.26	1.32
		Avoidance	1.55^∗∗^	0.45^∗∗^			0.70^∗∗^(0.18)	0.26	1.21
		Obstruction	0.44	0.19^∗∗^			0.08 (0.05)	–0.04	0.26
		Progress	–0.09	–0.21			0.00 (0.02)	–0.06	0.08
	Depression	Psy flex			0.68^∗∗^	0.32	0.36^∗∗^(0.13)	0.02	0.71
		Avoidance	1.55^∗∗^	0.15^∗∗^			0.24^∗∗^(0.08)	0.07	0.47
		Obstruction	0.44	0.25^∗∗^			0.11 (0.06)	–0.05	0.30
		Progress	–0.09	–0.15^∗∗^			0.01 (0.04)	–0.09	0.14
Anxiety	Pain interference	Psy flex			0.60^∗∗^	0.22	0.38^∗∗^(0.09)	0.17	0.61
		Avoidance	0.63^∗∗^	0.50^∗∗^			0.31^∗∗^(0.07)	0.14	0.51
		Obstruction	0.68^∗∗^	0.12			0.08 (0.05)	–0.06	0.23
		Progress	–0.40^∗∗^	0.02			−0.01(0.03)	–0.09	0.07
	Depression	Psy flex			0.73^∗∗^	0.54^∗∗^	0.18^∗∗^(0.05)	0.07	0.31
		Avoidance	0.63^∗∗^	0.15^∗∗^			0.09^∗∗^(0.03)	0.04	0.18
		Obstruction	0.68^∗∗^	0.07			0.05 (0.04)	–0.05	0.14
		Progress	–0.40^∗∗^	–0.11^∗∗^			0.04^∗∗^(0.02)	0.00	0.10

*All models adjusted for age. Psy flex, psychological flexibility. ^∗∗^*p* < 0.01.*

#### Direct and Indirect Effect of Pain Intensity on Pain Interference

The full model showed a significant indirect effect of psychological flexibility on the relationship between pain intensity and pain interference. Among the individual psychological flexibility factors only avoidance had a significant indirect effect in the full model.

In the analyses of each respective path avoidance illustrated significant coefficients in both the *a* and *b* paths. Of the value factors, obstruction showed significant *b* path.

#### Direct and Indirect Effect of Pain Intensity on Depression

A significant indirect effect was shown for the full model. Notably, the direct effect was not significant, which implies a strong indirect effect of psychological flexibility on the relation between pain intensity and depression. Again, avoidance individually showed a significant indirect effect in the full model. Furthermore, avoidance showed significance in both *a* and *b* paths, whereas value obstruction and value progress had significant *b* paths only.

#### Direct and Indirect Effect of Anxiety on Pain Interference

A significant indirect effect of psychological flexibility on pain interference was seen in the full model. The direct effect (anxiety on pain interference) was not significant, implying a strong indirect effect of psychological flexibility. Avoidance had an individually significant indirect effect in the full model. The analysis of individual paths showed avoidance had significant *a* and *b* paths in the model, where value obstruction and value progress showed significant *a* paths only.

#### Direct and Indirect Effect of Anxiety on Depression

The full model showed a significant indirect effect of psychological flexibility on depression. Among the individual psychological flexibility factors, both avoidance and value progress individually had significant indirect effect. Avoidance and value progress showed significant *a* and *b* paths while value obstruction only had significant *a* path.

## Discussion

The purpose of the present study was to examine the role and function of psychological flexibility – assessed with avoidance, value obstruction and value progress – as a resilience factor in a sample of 252 persons with chronic pain. In general, psychological flexibility was shown to be a significant factor in the relationship between symptoms (pain intensity and anxiety) and functioning (pain interference and depression).

Avoidance was shown to be particularly important for the influence of symptoms on functioning, as illustrated by significant *a*- and *b*-paths across all four models of indirect effects. The two value components, obstruction and progress, were primarily relevant in the association between anxiety and functioning, which warrants further studies to explore the relative importance of different aspects of psychological flexibility.

The results from the present study support research indicating the relevance of psychological flexibility in explaining variance in functioning in individuals with chronic pain (McCracken and Vowles, 2007; Zetterqvist et al., 2017). Previous studies have also shown that psychological flexibility is an important change mechanism in exposure-based interventions (Vowles et al., 2008, 2014; Wicksell et al., 2010b; Trompetter et al., 2015).

The conceptualization of resilience as a key factor in the relation between pain and functioning is seen also in a Scottish population-based study illustrating that resilient persons (high pain intensity and low disability score) had a higher 10-year-survival than vulnerable persons (low pain intensity, high disability score) (Elliott et al., 2014). In that study, factors associated with higher resilience were being male, lower age, higher education, owning your own home, and absence of chronic illness. Although these factors are informative of risk for higher disability, they are not directly modifiable to increase resilience in individuals with chronic pain, and thereby elevating functioning. The authors underline the importance of identifying modifiable factors (Elliott et al., 2014). Similarly, in a recent cross-sectional study (Richardson and Jost, 2019) on development of depression and PTSD following early life trauma, the authors emphasize the importance of evaluating psychological flexibility rather than traits or personal attributes as it “seems to be more adaptable to change and is an opportunity for therapeutic intervention.” This is also consistent with Goubert and Trompetter (2017) who emphasizes the importance of focusing on resilience factors that can be changed and used to improve the ability to “ward off, buffer against and recover from disability” for chronic pain patients. In the present study, resilience is conceptualized as a contextual behavioral factor, or a set of behaviors. This conceptualization is of particular clinical relevance, since (operant) behaviors are under contextual control, which implies they can be directly changed. Chronic pain has detrimental effects on functioning for many individuals. The results in the present study support the indirect effects of psychological flexibility – avoidance in particular – and suggest it as relevant target in treatment for chronic pain to improve functioning. This corresponds with previous research showing that avoidance is associated with functioning, and that addressing avoidance in exposure-based interventions can improve functioning (Wicksell et al., 2009; Vlaeyen et al., 2016; Bonnert et al., 2018; Hedman-Lagerlof et al., 2018). The results from the present study also supports the importance of values orientation, and the specific, or incremental, utility of interventions promoting value-oriented behaviors to improve resilience should be addressed in further research.

Furthermore, resilience is more than the absence of disability (Goubert and Trompetter, 2017), which corresponds with the conceptualization in the present study. Future research should further explore the construct of resilience by evaluating the importance of related variables among individuals with chronic pain and distress. For example, future research may benefit from using a longitudinal design to examine the mediating role of psychological flexibility as a resilience factor to allow for analyses of temporal relationships. Also, studies exploring the relationships between subprocesses of psychological flexibility, such as acceptance and present-moment-awareness, as well as other constructs relevant to the concept of resilience in chronic pain, are warranted and should ideally use behavioral measures such as task performance, in combination with self-report questionnaires. Lastly, experimental studies evaluating the effects of specific interventions on resilience are needed.

While the present study examines the role and function of psychological flexibility as a resilience factor in relation to chronic pain, it is worth noting the transdiagnostic properties of the psychological flexibility model, particularly as there is a need for psychological interventions that better meet the needs of patients with comorbid psychiatric and medical conditions (Barlow et al., 2004; Evans et al., 2005; Merikangas et al., 2007; Löwe et al., 2008). Psychological flexibility is not limited to chronic pain but a psychological skill, or set of skills, that has broad applicability and goes beyond any single mental or physical health condition (Dindo et al., 2017). Psychological *in*flexibility has been suggested to underlie a wide array of problems, including mental health, behavioral and comorbid complications (Hayes et al., 2006; Kashdan and Rottenberg, 2010). Psychological *in*flexibility has also been associated with mood- and anxiety disorders (Spinhoven et al., 2016). Conversely, improvements in psychological flexibility has been found to predict improvements in depressive symptoms in patients with borderline personality disorder (Berking et al., 2009), improvements in depression and anxiety in patients at risk for vascular disease (Dindo et al., 2015), and improvements in diabetes self-care, blood glucose levels and diabetes-related acceptance in patients with diabetes (Gregg et al., 2007) to mention a few. Although more and larger studies are needed, the empirical support including the present findings, suggest that psychological flexibility is an interesting and important resilience factor across conditions. To address the transdiagnostic nature of psychological flexibility future research should address if the level and implications of psychological flexibility varies across subgroups of patients, for example diagnoses and comorbidities.

In acceptance and commitment therapy (ACT) psychological flexibility, rather than a reduction in symptoms (for example pain and anxiety), is the key therapeutic target. However, ACT is an exposure-based treatment and share several important aspects with other forms of exposure therapy, such as graded exposure based on the fear-avoidance model. Future research should further explore the unique contribution of ACT-specific components such as acceptance and values-orientation, as well as differences and similarities in change processes between exposure-based treatments.

A few limitations should be considered when interpreting the results from the present study. The use of a cross-sectional design prevents any causal inferences. More research is needed to examine psychological flexibility as a resilience factor in longitudinal studies. Also, even though avoidance, value obstruction and value progress are variables relevant to resilience, other behavioral factors of potential importance that impact pain interference and depression such as sleep or social support, were not included. Furthermore, avoidance items from the Psychological Inflexibility in Pain Scale may have some conceptual overlap with pain interference (e.g., “I avoid scheduling activities because of my pain”). The levels of education as well as the proportion of women (81%) in the study sample is higher than the Swedish average, which may affect the generalizability of results. The sample is self-referred, which may imply limitations to the external validity. However, when compared with samples from a tertiary pain clinic the self-referred sample displays similar levels of pain, distress, and disability (Wicksell et al., 2008a, 2010b; Kemani et al., 2016).

## Conclusion

Psychological flexibility – in this study assessed as avoidance, value obstruction and value progress – plays a significant role as a resilience factor in the relationship between symptoms and functioning among individuals with chronic pain. Psychological flexibility has been successfully improved in previous clinical trials, and the present findings thus support the utility of this as an important target in treatment.

## Data Availability

The datasets generated for this study will not be made publicly available since the ethical permit does not allow sharing of data.

## Ethics Statement

Human Subject Research: This study was reviewed and approved by the Regional Board of Ethics, Stockholm, Sweden. All participants provided written informed consent prior to participation.

## Author Contributions

CG and JR collected the data. CG, JR, and RW designed the study. JR and VZ prepared the data and performed the preliminary data analysis. CG, JR, and RW performed the final data analyses. CG, JR, and RW prepared the manuscript with valuable contributions from ML, LS, and VZ. All authors approved the final version of the manuscript.

## Conflict of Interest Statement

The authors declare that the research was conducted in the absence of any commercial or financial relationships that could be construed as a potential conflict of interest.
